# Supplementary material for: 1H NMR studies distinguish the water soluble metabolomic profiles of untransformed and RAS-transformed cells
Source: PeerJ. 2016 Jun 7;4:e2104. doi: 10.7717/peerj.2104 (PMC4906648; doi:10.7717/peerj.2104)
Supplement: Table S1 — The adjusted p-values from post-hoc/multiple comparison testing of the effective NMR metabolite fractions ((1) in the main text) using the Benjamini Yekutieli (BY) algorithm (Benjamini & Yekutieli, 2001) with a false discover rate of 0.01. Only those metabolites where an ANOVA analysis indicated that the average value of NMR metabolite fraction was different between at least two of the four cell types are listed. Significant differences (adjusted p-values ≤0.01) are bolded in red. [file peerj-04-2104-s001.docx]

| $x_{metabolite}$ | *p*(control,NRAS) | *p*(control,KRAS) | *p*(control,HRAS) | *p*(NRAS,KRAS) | *p*(NRAS,HRAS) | *p*(KRAS,HRAS) |
| --- | --- | --- | --- | --- | --- | --- |
| Alanine | **1.4 x 10^-7^** | **4.7 x 10^-3^** | **3.4 x 10^-5^** | 8.3 x 10^-2^ | 4.1 | 7.4 x 10^-2^ |
| Aspartate | 3.4 | **1.2 x 10^-4^** | 0.31 | **6.7 x 10^-4^** | 0.61 | 0.64 |
| Choline | **1.2 x 10^-5^** | **9.2 x 10^-6^** | **3.1 x 10^-5^** | 4.7 | 3.9 | 3.0 |
| Choline  Alfoscerate | 1.7 x 10^-2^ | 5.2 | 0.32 | **1.1 x 10^-5^** | 0.20 | 5.5 x 10^-2^ |
| Creatine | 1.8 x 10^-2^ | **3.9 x 10^-3^** | 2.1 x 10^-2^ | 1.1 | 3.0 | 4.5 |
| Fumarate | **2.4 x 10^-3^** | **3.1 x 10^-4^** | **4.4 x10^-3^** | 3.4 | 1.6 | 0.22 |
| Lactate | 1.4 x 10^-2^ | **3.7 x 10^-3^** | 1.2 x 10^-3^ | 5.0 | 1.5 | 1.5 |
| Leucine | 1.1 x 10^-2^ | 0.10 | **6.5 x 10^-3^** | 3.1 | 0.33 | 0.18 |
| Myo-inositol | **6.0 x 10^-10^** | **1.2 x 10^-6^** | **5.6 x 10^-9^** | **3.7 x 10^-3^** | 1.9 | 1.1 x 10^-2^ |
| NAA | **3.7 x 10^-3^** | **4.0 x 10^-3^** | 1.3 | 4.1 | 0.26 | 0.37 |
| NAC | **2.5 x 10^-4^** | **5.9 X 10^-4^** | **4.1 X 10^-4^** | 0.61 | **7.9 x 10^-3^** | 0.30 |
| Phe | **3.3 x 10^-6^** | 2.7 x 10^-2^ | 0.11 | 0.35 | 5.0 | 1.6 |
| PC | **8.1 x 10^-7^** | **1.7 x 10^-6^** | **8.1 x 10^-7^** | **2.9 x 10^-8^** | **2.5 x 10^-4^** | **1.9 x 10^-5^** |
| Proline | **1.8 x 10^-4^** | **1.2 x 10^-4^** | 2.7 x 10^-2^ | 4.2 | 4.3 x 10^-2^ | 2.1 x 10^-2^ |
| Taurine | **6.0 x 10^-10^** | **1.0 x 10^-6^** | **7.3 x 10^-7^** | 1.5 x 10^-2^ | 4.7 | 3.5 x 10^-2^ |
| Tyrosine | **6.5 x 10^-3^** | 4.7 x 10^-2^ | **8.2 x 10^-4^** | 1.0 | 1.8 x 10^-2^ | 1.5 x 10^-2^ |
| UDP-X | **1.4 x 10^-7^** | **6.9 x 10^-3^** | **4.1 x 10^-3^** | 9.0 x 10^-2^ | **1.0 x 10^-5^** | 0.2’6 |
| Valine^##^ | 0.93 | 1.9 | 2.5 x 10^-2^ | 4.1 | 0.11 | 8.6 x 10^-2^ |

**Table S1**
