# Supplementary material for: 1H NMR studies distinguish the water soluble metabolomic profiles of untransformed and RAS-transformed cells
Source: PeerJ. 2016 Jun 7;4:e2104. doi: 10.7717/peerj.2104 (PMC4906648; doi:10.7717/peerj.2104)
Supplement: Table S2 — The adjusted p-values from post-hoc/multiple comparison testing of the glutamate normalized signals, (Eq. (3) in the main text) using the Benjamini Yekutieli (BY) algorithm (Benjamini & Yekutieli, 2001) with a false discover rate of 0.01. Only those metabolites where an ANOVA analysis indicated that the average value of glutatmate normalized signal was different between at least two of the four cell types are listed. Significant differences (adjusted p-values ≤0.01) are bolded in red. [file peerj-04-2104-s002.docx]

| $\xi_{metabolite,glutamate}$  **Table S2** | *p*(control,NRAS) | *p*(control,KRAS) | *p*(control,HRAS) | *p*(NRAS,KRAS) | *p*(NRAS,HRAS) | *p*(KRAS,HRAS) |
| --- | --- | --- | --- | --- | --- | --- |
| Alanine | **6.0 x 10^-4^** | **4.5 x 10 ^-3^** | 3.3 x 10^-2^ | 0.55 | 3.3 | 0.71 |
| Beta-alanine | 7.5 x 10^-2^ | **2.6 x 10^-4^** | 9.4 x 10^-2^ | 1.3 | 4.1 | 0.61 |
| Aspartate | 2.5 | **2.6 x 10^-4^** | 0.12 | **2.4 x 10^-4^** | 0.29 | 0.47 |
| Choline | **6.1 x 10^-3^** | **2.6 x 10^-4^** | 3.0 x 10^-2^ | 5.1 | 3.4 | 3.3 |
| Choline  Alfoscerate | 2.1 x 10^-2^ | 4.8 | 0.44 | **3.8 x 10^-4^** | 1.4 | 0.29 |
| CP^##^ | 7.1 x 10^-2^ | 0.12 | 0.21 | 2.8 | 1.9 | 3.4 |
| Fumarate | **1.2 x 10^-3^** | **2.3 x 10^-4^** | **2.4 x 10^-3^** | 2.4 | 1.9 | 0.21 |
| Glutamine^##^ | 1.6 | 1.1 x 10^-2^ | 0.20 | 1.7 x 10^-2^ | 0.96 | 1.0 x 10^-2^ |
| Myo-inositol | **1.9 x 10^-7^** | **1.4 x 10^-5^** | **2.4 x 10^-6^** | **1.6 x 10^-3^** | 1.6 | 6.7 x 10^-2^ |
| NAA | **1.1 x 10^-3^** | 1.3 x 10^-2^ | 3.2 | 2.8 | 0.33 | 0.67 |
| NAC | **3.7 x 10^-4^** | **6.3 x 10^-3^** | 1.7 x 10^-2^ | 0.78 | 1.8 x 10^-2^ | 0.55 |
| PC | **1.5 x 10^-7^** | **6.4 x 10^-8^** | **3.6 x 10^-8^** | **2.3 x 10^-7^** | **7.1 x 10^-4^** | **4.7 x 10^-5^** |
| Proline | **6.0 x 10^-4^** | **1.4 x 10^-4^** | 0.29 | 3.2 | 1.1 | 0.56 |
| Taurine | **2.5 x 10^-7^** | **1.4 x 10^-6^** | **1.5 x 10^-7^** | **1.8 x 10^-3^** | 5.0 | **3.4 x 10^-3^** |
| Tyrosine | 1.6 x 10^-2^ | 8.3 x 10^-2^ | **2.2 x 10^-3^** | 1.5 | 0.35 | 0.11 |
| UDP-X | **3.6 x 10^-8^** | **5.3 x 10^-3^** | **3.4 x 10^-3^** | 0.13 | **1.4 x 10^-5^** | 0.29 |

**Benjamini Y, Yekutieli D. 2001.** The control of the false discovery rate in multiple testing under dependency. *The Annals of Statistics* **29(4)**: 1165-1188.
