# Supplementary figures and images for: 1H NMR studies distinguish the water soluble metabolomic profiles of untransformed and RAS-transformed cells
Source: PeerJ. 2016 Jun 7;4:e2104. doi: 10.7717/peerj.2104 (PMC4906648; doi:10.7717/peerj.2104)

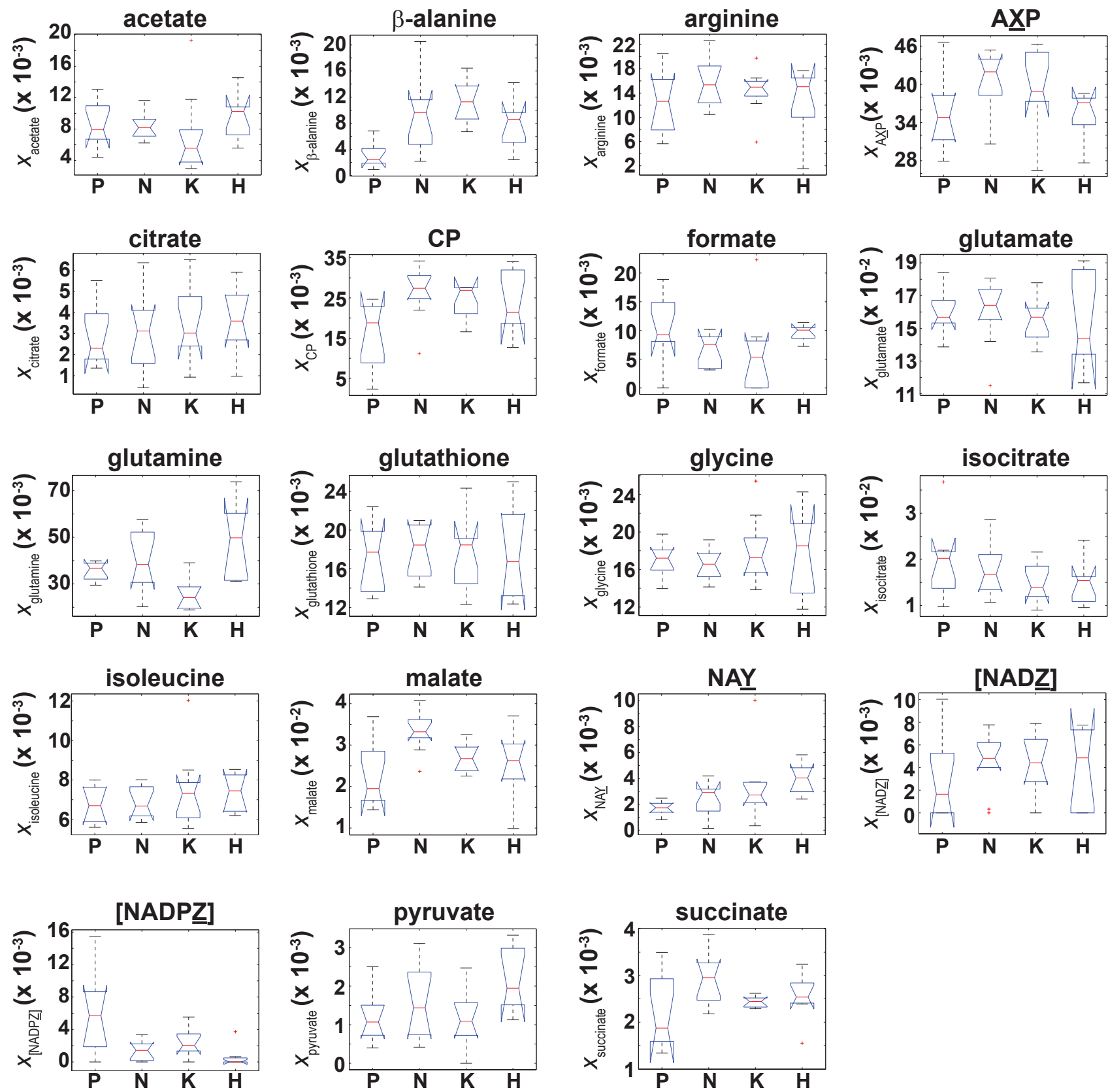

Supplement: Figure S1 — Box plots of NMR metabolite fraction for those 19 metabolites where an ANOVA test did not identify a significant difference in the average NMR metabolite fraction between at least two of the four cell types. [file peerj-04-2104-s003.pdf]

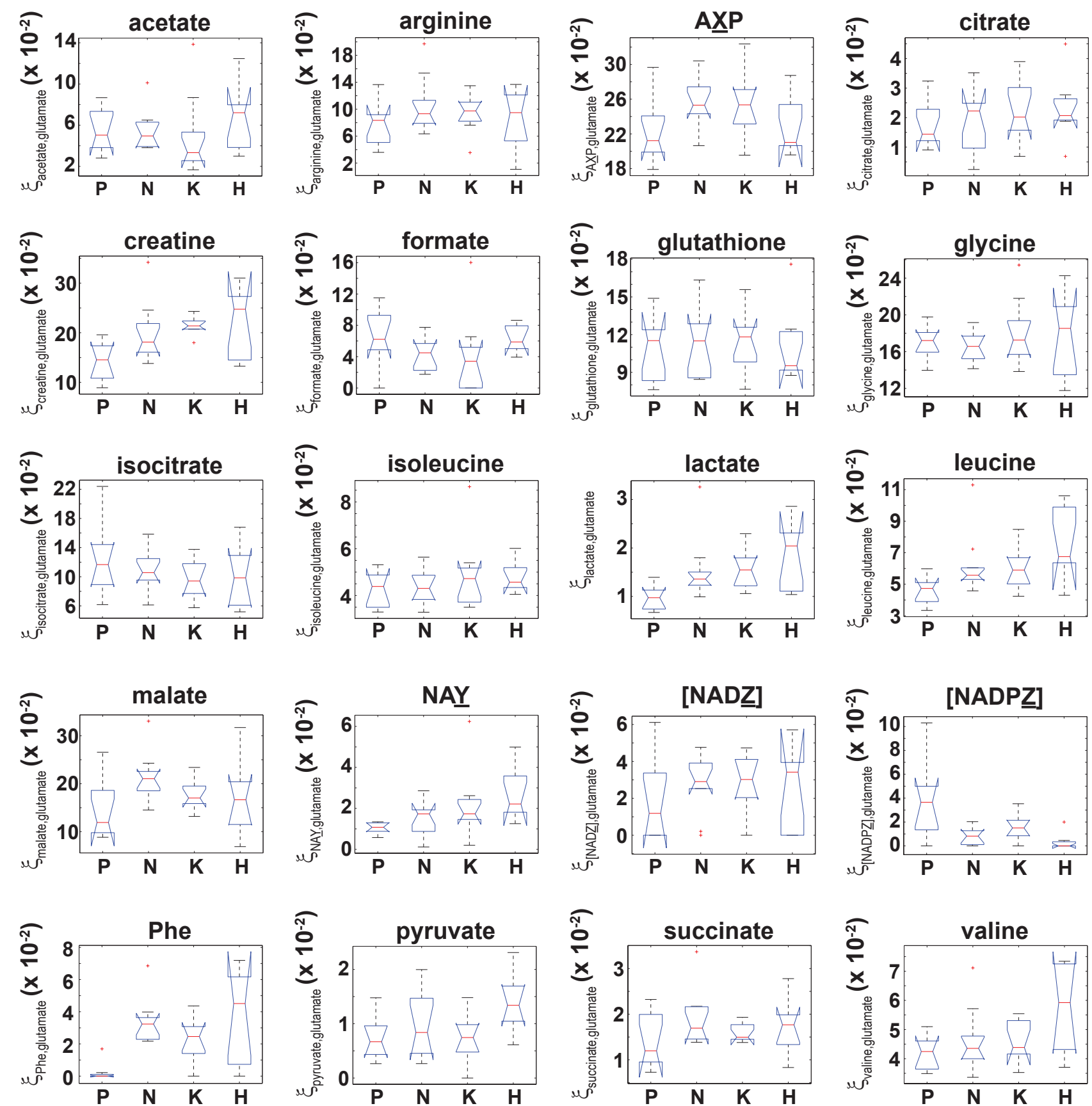

Supplement: Figure S2 — Box plots of the glutamate normalized signals for those 20 metabolites where an ANOVA test did not identify a significant difference in the average glutamate normalized signal between at least two of the four cell types. [file peerj-04-2104-s004.pdf]
